# EV‐LYSG101: Extracellular Vesicles Loaded With Lysin LYSG101 for Potent Anti‐Staphylococcal Activity

**DOI:** 10.1002/jex2.70174

**Published:** 2026-08-06

**Authors:** Dominique B. Hoelzinger, Ava M. Koehler de Celaya, Cheryl E. Myers, Natasha P. Dyal, Christopher F. Saling, Raymond Schuch, Gina A. Suh

**Affiliations:** ^1^ Department of Regenerative Biotherapeutics Mayo Clinic Scottsdale Arizona USA; ^2^ Division of Infectious Diseases Mayo Clinic Phoenix Arizona USA; ^3^ Precisio Biotix Therapeutics, Inc. Dover Delaware USA; ^4^ Division of Public Health, Infectious Diseases, and Occupational Medicine, Department of Medicine Mayo Clinic Rochester Minnesota USA

**Keywords:** antimicrobial, extracellular vesicles, lysin, LYSG101, MRSA, prosthetic joint infection, *Staphylococcus aureus*

## Abstract

Biofilm‐associated staphylococcal infections remain exceptionally difficult to treat due to the presence of resilient staphylococcal biofilms and the limited effectiveness of currently available antibiotics. To combat persistent infections, providers are often forced to use prolonged courses of combination antimicrobial therapies that have significant toxicities with limited effectiveness, leading to increased hospital stays, substantial health care costs and a rise in patient morbidity and mortality. *Staphylococcus aureus* is a dominant cause of recalcitrant biofilms, for which novel non‐antibiotic therapeutics are critically needed. Lysins, a class of protein‐based antimicrobials, rapidly kill staphylococci, exhibit potent anti‐biofilm activity, have a low propensity of resistance development and synergy with antibiotics. To address this unmet need, we evaluate an extracellular vesicle (EV)‐based delivery platform for the engineered anti‐staphylococcal lysin LYSG101, designed to improve stability and localization at sites of infection. Initial proof‐of‐concept work is provided here, demonstrating that human serum‐derived EVs can be loaded with LYSG101 to exert a potent in vitro antimicrobial effect against both planktonic and biofilm forms of *S. aureus*. EV‐mediated delivery achieved activity equivalent to free lysin, with additional translational advantages including stability and the potential for sustained intra‐articular retention. This work supports further development of the EV‐mediated lysin delivery as a broadly applicable antimicrobial platform, with potential future applications in biofilm‐associated infections, including prosthetic joint infection (PJI).

## 1 Introduction

Biofilm‐associated staphylococcal infections remain exceptionally difficult to treat because bacteria embedded within biofilm structures exhibit reduced susceptibility to antibiotics and host immune clearance. These infections occur across a range of clinical settings, including prosthetic joint infection (PJI), osteomyelitis, endocarditis, and infections involving indwelling medical devices. Despite advances in antimicrobial therapy and surgical management, treatment failure rates remain substantial, highlighting the need for new therapeutic strategies capable of targeting biofilm‐associated bacteria.

Lysins are a promising new family of enzyme (cell wall hydrolase)‐based antimicrobials derived from bacteriophage. Therapeutic use of lysins was first proposed based on studies showing that recombinantly produced protein can target bacteria for rapid killing, in a process called ‘lysis from without’ (Fischetti et al. 2006; Loeffler et al. 2001; Nelson et al. 2001; Schuch et al. 2002). Lysins have been evaluated against a range of human pathogens, both in vitro and in a wide variety of animal infection models, demonstrating efficacy and safety and elucidating features that include rapid cidality, target specificity (i.e. narrow spectrum) and, importantly, anti‐biofilm activity (Defraine et al. 2016; Indiani et al. 2019; Karau et al. 2023; Sauve et al. 2024; Schuch et al. 2017, 2022; Vila‐Farres et al. 2023; Watson et al. 2019).

Lysins are, in particular, well suited to eradicate antibiotic resistant bacteria embedded in biofilms and are envisioned for clinical applications in osteomyelitis (Karau et al. 2019, 2022; Keller et al. 2023), PJI (Ferry et al. 2021; Sosa et al. 2020; Souche et al. 2022), endocarditis (Indiani et al. 2019; Kebriaei et al. 2021; Shah et al. 2020) and pneumonia (Loeffler et al. 2001; Sauve et al. 2024; Alreja et al. 2024; Swift et al. 2021). The antibiofilm effect is intriguing, as there are no FDA‐approved antibiotics to eradicate biofilms and eliminate sources of chronic infection. Lysins also exhibit low resistance development, no antibiotic cross‐resistance and synergy with antibiotics (Gerstmans et al. 2016; Oh et al. 2019, 2023; Schuch et al. 2014; Watson et al. 2020). These features underpin interest in lysins as therapeutics, which are structurally and functionally distinct from antibiotics.

Despite promising preclinical results, clinical development of lysins remains limited by unresolved challenges related to stability, immunogenicity and delivery (Schuch et al. 2022; Cui et al. 2025; Murray et al. 2021). Two lysins have reported clinical data—exebacase and LSVT‐1701, both for intravenous delivery in staphylococcal bacteremia and endocarditis. After promising findings in Phase 1 and Phase 2, the exebacase DISRUPT trial was halted in Phase 3 because of efficacy concerns (Fowler et al. 2020, 2024). LSVT‐1701 demonstrated favourable safety in Phase 1, however, the trial was halted in Phase 2 (NCT03089697) for ‘strategic reasons’ (Wire et al. 2022). More promising findings were reported for exebacase administered intra‐articularly in compassionate use for patients with recalcitrant PJI to prevent major loss of function (Ferry et al. 2021). For applications such as PJI, administration directly at sites of infection, as with PJI, offers distinct advantages over systemic (i.v.) administration, in particular with respect to concerns of proteolytic degradation and limited bioavailability.

Lysin stability is influenced by factors including temperature, pH, and ionic strength, and many lysins are prone to poor activity under physiological conditions (Cui et al. 2025). To address this and to facilitate clinical translation, lysins can be engineered for stability and potent activity in relevant conditions (i.e. plasma, synovial fluid, respiratory fluid), as recently described for anti‐staphylococcal lysin, LYSG101 (formerly called ClyO [Xiao et al. 2026]). LYSG101 is a chimeric lysin comprised of the catalytic domain of Ply187 (Loessner et al. 1999) and the binding domain of PlySS2 (Gilmer et al. 2013) as described (Xiao et al. 2026), that exhibits many of the promising features of the lysin family, is rapidly bactericidal and exhibits anti‐biofilm activity against all isolates of *Staphylococcus aureus* and coagulase‐negative staphylococci in physiological testing matrices. As an engineered lysin, LYSG101 is designed to overcome the translational hurdles seen with progenitors such as exebacase.

In addition to the engineering of LYS101, we are facilitating clinical translation by developing delivery strategies that enhance stability and localization of lysin therapeutics for biofilm‐associated infections, with PJI representing one important potential application. Such approaches have been described for lysins, which include PEGylation (Resch et al. 2011), protein fusions (Resch et al. 2011; Kaur et al. 2020) and nanocarriers (Kaur et al. 2020). The use of extracellular vesicles (EVs) is of particular interest for lysins (Lee et al. 2025). Research into EVs for drug delivery is expanding because of properties related to low immunogenicity, homing capacity, stability, sustained release and safety (Rakshit and Pal 2024; Jeppesen et al. 2023; Witwer and Wolfram 2021). Multiple techniques for loading protein and nucleic acid cargo into EVs have been developed (Busatto et al. 2021; Luo et al. 2021; McAndrews et al. 2021). Intravenous (i.v.) delivery of loaded EVs in mouse models shows rapid accumulation in the liver, spleen, and lung, and persistence for over 24 h (Gerwing et al. 2020; Wiklander et al. 2015), providing sustained release of cargo in these organs. More recent studies of EV distribution in a non‐human primate model showed that i.v. dosing of fluorescently labelled EVs accumulated in liver, spleen, kidney and lung (Driedonks et al. 2022). In addition, they demonstrated that granulocytes, monocytes, B‐ and T‐cells from peripheral blood took up labelled EVs, suggesting that therapeutic cargo could be widely disseminated throughout the body. For usage in PJI, localized delivery is preferred due to the extended retention of EVs inside the joint space. Intra‐articular injection of human mesenchymal stem cell‐derived (MSC) EVs in rodent models of osteoarthritis has shown retention in the joint capsule and sustained release of anti‐inflammatory and regenerative activities in the joint (Gupta et al. 2024; Liang et al. 2022; Wang et al. 2017; Zhao et al. 2023; Zhou et al. 2022). Expansion of EVs for delivery of antibiotics and antimicrobial peptides is of great interest (Heinrich et al. 2023; Ibrahim et al. 2024), with the additional benefit of targeting intracellular staphylococci (Yuan et al. 2025). Intracellular *Staphylococcus* can serve as a reservoir for chronic PJI, evading host defenses and antibiotics by persisting within osteoblasts and osteocytes (Heckmann et al. 2025). As a delivery system for LYSG101, EVs would thus have the added benefit of targeting intracellular bacteria, in addition to biofilm and planktonic forms.

In this study, we describe development of an EV‐based delivery strategy for the engineered lysin LYSG101 as a potential platform to enhance stability and localization of lysin therapeutics. Although PJI represents one clinically relevant application motivating this work, the primary objective of this study was to establish proof‐of‐concept feasibility of EV loading and preservation of antimicrobial activity following encapsulation. Proof‐of‐concept studies are described herein, with the description of EV purification and efficient loading with LYSG101. The resulting EV‐LYSG101 compound is stable in a lyophilized form and, in suspension, exerts a potent anti‐staphylococcal effect, in particular against *S. aureus* biofilms. Further development of the EV‐LYSG101 modality is thus warranted.

## 2 Materials and Methods

### 2.1 Bacterial Strains and Isolates

Bacteria used in this study are as follows: (1) methicillin‐sensitive *S. aureus* (MSSA) ATCC 29213, (2) methicillin‐resistant *S. aureus* (MRSA) ATCC 43300, (3) MRSA strain ATCC BAA‐42 (ATCC, Manassas, VA), (4) methicillin‐resistant *S. epidermidis*, three different human clinical isolates from Mayo Clinic, (5) *Escherichia coli* strain ATCC 15922. The engineered anti‐staphylococcal lysin LYSG101 (28.6 kDa) was provided by Presicio Biotix Therapeutics, Inc. (Dover, DE) as pure protein in 20 mM phosphate buffer pH 7.4, produced as described (Xiao et al. 2026).

### 2.2 EV Source and Purification

MISEV guidelines (Welsh et al. 2024) for best practices in EV isolation were observed throughout the steps of preparing and validating EVs. Fifty millilitres of human peripheral blood was collected from 12 healthy donors (IRB‐22‐009973 [Mayo Clinic]) in serum Vacutainer tubes (BD 367820). There were four males and eight females ranging in age from 23 to 67 years old (average 49.4 years). Tubes were incubated at room temperature for 30 min and then centrifuged at 2000 × *g* for 20 min. Serum was collected and stored in 5 mL aliquots at ‐80°C.

EVs were aseptically isolated using size exclusion chromatography (SEC) with a 70 nm cutoff cartridge (qEV1 or qEV10; Izon Science Ltd., Christchurch, New Zealand) and an automatic fraction collector (Izon Science Ltd.) in a manner similar to that previously described (Vanderboom et al. 2021). The EVs were diluted 1:10 in sample diluent (PBS with 1% TWEEN passed through 0.2 µm filter), measured and quantitated on a C‐400 chip using microfluidic resistive pulse sensing technology (MRPS) in a nCS2 particle analyzer (Spectradyne, Signal Hill, CA), counting > 500 particles with a statistical error in concentration of <5%. EVs were stored in phosphate‐buffered saline supplemented with 25 mM trehalose (PBS‐T, filtered by passage through a 0.22 µm filter), aliquoted and stored at ‐80°C. Induced pluripotent stem cell (iPSC) EVs were acquired from Applied StemCell Inc. (Milpitas, CA) and bone marrow‐derived mesenchymal stem cell (BM‐MSC) EVs were acquired from Rooster Biologics (Frederick, MD).

### 2.3 Western Blots

Peripheral blood mononuclear cells (PBMCs) and EVs were resuspended in 1× sample buffer (ProteinSimple, San Jose, CA) and protein concentration was measured using the Pierce BCA protein assay kit (ThermoFisher Scientific, Parsippany, NJ). Protein (20 µg) was denatured and resolved by electrophoresis using Mini‐Protean TGX 4%–20% gradient gels (BioRad, Hercules, CA) and then transferred to polyvinylidene difluoride (PVDF) membranes using the Transblot Turbo transfer system and reagents (BioRad). Total protein per lane was quantitated using Revert 700 Protein Stain (Li‐Cor Inc., Lincoln, NE). Membranes were probed with antibodies for Calnexin (CANX Novus NBP1‐47546, Novus Biologicals, Littleton, CO), CD63 (Novus, NBP2‐42225), CD9 (Cell Signaling Technologies, 13174, Danvers, MA) and syndecan binding protein (SDCBP, Syntenin 1, Novus, NBO2‐50287) and visualized with the corresponding secondary IRDye 680 and IRDye 700 antibodies (Li‐Cor Inc.) following manufacturers’ instructions and imaged with the Odyssey CLx (Li‐Cor Inc.).

### 2.4 LYSG101 Loading Into EVs

Up to 1E11 EVs were loaded with 16 µg LYSG101 by electroporation using a dibasic phosphate electroporation buffer (DPEB: Ficoll 21%, dibasic potassium phosphate 1.15 mM, potassium chloride 25 mM in H_2_O) based on Lamichhane et al., 2015 (Lamichhane et al. 2015). Briefly, 1E11 EVs were filter concentrated using Amicon 100 kDa filters (UFC510096, MilliporeSigma, Burlington, MA) to a volume of 25–50 µL, LYSG101 was then added to desired concentration, and DPEB was added to 400 µL. Samples were placed in a 0.4 cm Gene Pulser Cuvette (BioRad) and subjected to 2 × 100 V square wave pulses of 25 ms each. For fewer EVs, optimal loading parameters were: 1E9‐1E10 EVs in 25 µL PBS, LYSG101 was added to the desired concentration and DPEB was added to 200μ l in a 0.2 cm Gene Pulser Cuvette; using 2 × 300 V exponential decay pulses. Following electroporation, EVs were washed twice with PBS using 100 kDa cutoff Amicon filters, resuspended in 200 µL, counted and sized a second time with the nCS2 and stored at –80°C. There routinely was a one‐order‐of‐magnitude decrease in EV‐LYSG101 numbers after loading; however, this allowed for the quantification of the number of EVs used in each experiment. The first flow‐through of the wash step was reserved for ELISA assays.

### 2.5 Flow Cytometry

EV loading with LYSG101 was visualized by fluorescently labelling the lysin with AF488 fluorophore (Alexa Fluor 488 Microscale Protein Labeling Kit, Invitrogen) following the manufacturers' instructions, ensuring that unincorporated dye was removed with the provided resin. LYSG101‐AF488 was loaded into 1E9 EVs by electroporation with excess lysin removed by two washes with PBS and filter concentration using Amicon Ultra 100 kDa 0.5 mL centrifugal filters. Loaded EVs (EV‐LYSG101‐AF488) were counterstained with Cell Trace Far Red (CTFR, Invitrogen), which crosses the plasma membrane and covalently binds to free amines on and inside the cells, to ensure visualization of EV‐associated LYSG101‐AF488 and not merely external aggregates. Additional controls included filtered water only, unstained EVs and single stained EVs. Observing MiFlowCyt‐EV (Welsh et al. 2020) guidelines, EV‐LYSG101‐AF488 were analysed by flow cytometry (Aurora, Cytek Biosciences, Fremont, CA) using 0.1 µm filtered water and a 0.04 µm in‐line filter (PureFlo Capsule, BioProcess Solutions, Bedford, Massachusetts).  Apogee 1572 fluorescent beads (Apogee Flow Systems, Hemel Hempstead, UK) were used to set the cytometer parameters for detection of sub‐micron particles. Gains for EVs were: FSC‐H 100, SSC‐H 50, thresholds were: FSC‐H 4000, SSC‐H 3500. Particles were gated on CTFR, and AF488 (Figure S1) intensities were measured as a reporter for levels of LYSG101 present in EVs.

### 2.6 Enzyme‐Linked Immunosorbent Assay (ELISA)

Loaded and quantified EV‐LYSG101 as described in Methods Section 2.4, were used to evaluate levels of incorporated LYSG101. Samples were prepared for ELISA as follows: (1) 10 µL of post‐wash flow‐through (FT) to measure unincorporated LYSG101, (2) 10 µL of EV‐LYSG101 mixed with 1 µL of 10× sample buffer (ProteinSimple, San Jose, CA) to measure lysin inside and on the EV surface (lysed), and (3) 10 µL of intact EVs to measure LYSG101 on the surface of EVs (whole). Each of these samples was diluted 1:20 in PBS, and 100 µL (containing 6.2E6 ± 2E5 EV‐LYSG101) was applied per well. Levels of LYSG101 were measured by sandwich ELISA using Immulon 4HBX flat bottom 96 well plates (3855, ThermoFisher Scientific), proprietary affinity‐purified polyclonal goat anti‐LYSG101 capture and affinity‐purified polyclonal rabbit anti‐LYSG101 detector antibodies (produced for Precisio Biotix Therapeutics by ZoonBio Biotechnology, Nanjing, China) using the following steps: (1) Plates were coated in goat anti‐LYSG101 2 µg/mL in coating buffer (100 mM sodium carbonate and 35 mM sodium bicarbonate, pH 9.5) overnight at 4°C, with three washes of TBS‐T. (2) Plates were blocked with KPL 10% BSA Diluent/Blocking Solution kit (SeraCare, Gaithersburg, MD) for 1 h at room temperature, with three washes of TBS. (3) Samples were resuspended in diluent, incubated at room temperature for 1 h and washed with PBS. (4) Rabbit anti‐LYSG101 detector antibody was used at 2 µg/mL in diluent for 30 min at room temperature, then washed four times in TBS‐T. (5) Goat anti‐rabbit Biotin (AB97049, Abcam, Cambridge, UK) 1:20,000 in diluent for 30 min at room temperature with four washes of TBS‐T. (6) Streptavidin HRP (AB7403, Abcam) 1:30,000 in diluent for 30 min at room temperature with four washes of TBS‐T. (7) 100 µL TMB ELISA substrate high sensitivity (AB171523, Abcam) was added for 2–3 min. (8) The enzymatic reaction was stopped with 1% HCl. (9) Plates were read on a SpectraMax M3 plate reader (Molecular Devices, San Jose, CA) using the TMB protocol.

### 2.7 Scanning Electron Microscopy (SEM)

Fresh MSSA colonies grown overnight on blood agar plates (Remel Inc., San Diego, CA) were picked and resuspended in PBS to a turbidity of 0.5 McFarland units, diluted 1:250 in tryptic soy broth with 0.2% glucose (TSBg), and 1 mL was aliquoted per well in a 24‐well flat bottom plate containing silicon wafers (catalog number 16006, Ted Pella Inc, Redding, CA) and grown for 18 h at 37°C. Wafers were then washed twice with 2 mL DPBS/Ca^+^Mg^+^ (gibco 14040–133) and incubated 1 h at room temperature under four conditions in the presence of the MSSA strain: (1) no additive (nil), (2) EVs (5E10 native EVs [no lysin]), (3) 25 ng/mL LYSG101 (no EVs) and (4) EV‐LYSG101 (5E10 EV‐LYSG101). Wafers were washed twice with PBS and fixed in a solution containing 2.5% glutaraldehyde (cat. 16020, Electron Microscopy Sciences, Hatfield, PA) in 0.06 M phosphate buffer, pH 7.2 (4.68 g monobasic sodium phosphate S3139, and 5.58 g dibasic sodium phosphate, Millipore‐Sigma, in 1 Lt ddH_2_O) for 90 min at room temperature. Following fixation, samples were washed four times for 10 min each in phosphate buffer to remove residual fixative. Dehydration was performed through a graded acetone series (Sigma‐Aldrich, cat. SHBS3485) with two sequential 10 min incubations at each concentration (50%, 70%, 90% and 100%). After complete dehydration, samples were processed using a Critical Point Dryer (Leica EM CPD300, Teaneck, NJ) and dried specimens were subsequently sputter‐coated with a 4 nm layer of gold‐palladium alloy to enhance conductivity. Scanning electron microscopy was performed using a Phenom PRO X Desktop SEM (Thermo Fisher Scientific) equipped with software version 6.8.4.rel.e2fd11083c.28328. Images were acquired under standard operating conditions and analysed for morphological characterization. Intact and lysed cells were counted from five regions of interest per sample (at 1000× magnification), tabulating percent intact cells.

### 2.8 EV‐LYSG101 Lytic Activity

Bacterial strains were grown on blood agar plates (Remel, Inc.) for 18 h, followed by resuspension in PBS/Ca^+^Mg^+^ at a turbidity equivalent of 9.5 McFarland units (OD _600_ = 0.5) (unless otherwise indicated). Measurements were performed using a Photometer DEN 600 (Grant‐Bio, Elmwood Park, NJ). EV‐LYSG101 preparations were diluted 1:4 in PBS/Ca^+^Mg^+^, and 100 µL of this preparation was co‐cultured with 100 µL of the *S. aureus* or *S. epidermidis* sample prepared as above in a 96‐well culture plate. The in vitro kinetic lysis assay was performed in a manner similar to that described (Gerstmans et al. 2016). Fresh cultures were incubated alone or with serial dilutions of LYSG101, EVs or EV‐LYSG101 in triplicate and incubated at 37°C, measuring OD_600_ every 15 min for 4 h on a SpectraMax M3 plate reader (Molecular Devices).

### 2.9 EV‐LYSG101 Biofilm Eradication

*S. aureus* strain ATCC BAA42 was used based on its robust in vitro production of biofilm (Schuch et al. 2017). Fresh colonies were grown overnight on blood agar plates (Remel, Inc.), individual colonies were picked and resuspended in PBS at a turbidity equivalent of 0.5 McFarland units, and diluted 1:150 in TSBg. Aliquots of 100 µL were then added per well to a 96‐well flat bottom plate and grown for 24 h at 37°C. Biofilm was confirmed by microscopy; wells were washed twice with 200 µL PBS, followed by the addition of the dilution series of LYSG101, EVs or EV‐LYSG101 in triplicate. Plates were incubated at 37°C and OD_600_ was read every 30 min for 5 h using a SpectraMax M3 plate reader (Molecular Devices). Biofilm eradication was also confirmed microscopically. Viable bacteria remaining after treatment were determined by quantitative plating and by measuring absorption at OD_600_ with a SpectraMax M3 plate reader.

### 2.10 Lyophilization of EV‐LYSG101

EV‐LYSG101 (6E11 particles, loaded with 16 µg LYSG101) in 2.4 mL PBS/Ca^+^Mg^+^ were aliquoted into 24 tubes containing 100 µL each: 12 tubes were frozen and stored at −20°C, 12 tubes were lyophilized (Labconco Freezone 6 Plus; Labconco Corporation, Kansas City, MO) for 12 h and stored at room temperature for up to 6 months. At indicated time points, samples were reconstituted as follows: 300 µL PBS/Ca^+^Mg^+^ was added to the frozen samples, the freeze‐dried samples were first rehydrated with 100 µL sterile water, and then 300 µL PBS/Ca^+^Mg^+^ was added to bring both samples to a volume of 400 µL. Lytic activity was tested in triplicate, measuring OD_600_ every 15 min for 3 h. Each well contained approximately 2E9 EV‐LYSG101 with 230 ng encapsulated LYSG101.

### 2.11 Statistical Analyses

All results are displayed as means ± standard deviations.

## 3 Results

### 3.1 EV Purification and Characterization

**FIGURE 1:**
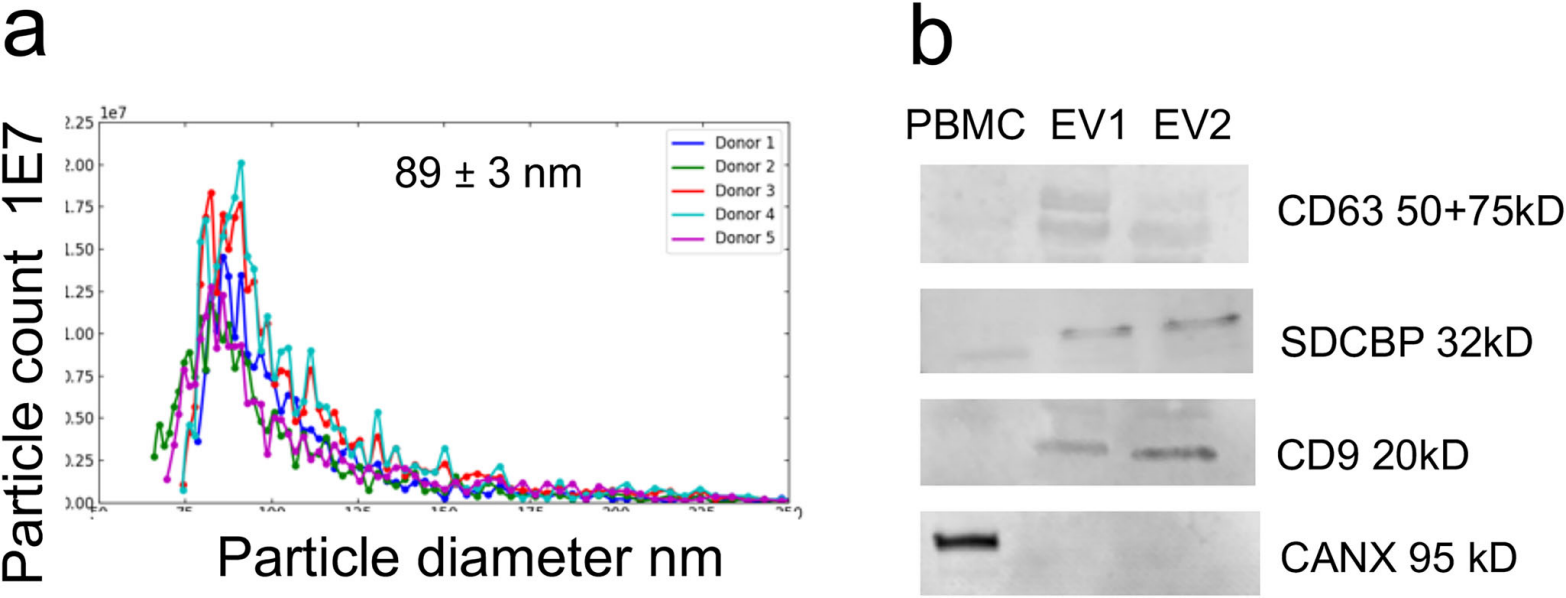
Characterization of human serum‐derived EV particles. (a) Quantification and sizing of EV isolates from five donors with microfluidic resistive pulse sensing (nSC2, Spectradyne) show an average diameter of 89 ± 3 nm. (b) Western analysis showing expression of EV markers CD9, CD63 and SDCB in EVs and not in peripheral blood mononuclear cells (PBMC), whereas CANX is expressed uniquely in PBMC. Representative data from two independent determinations.

Aseptically isolated EVs from individual donor serum were measured and quantitated using MRPS, yielding approximately 3E11 EVs/mL of serum with a consistent size distribution averaging 89 nm ± 3 nm in diameter (Figure 1a). Preparations were evaluated by western blot to confirm expression of proteins known to be associated with EVs, such as CD63, CD9 and syndecan binding protein (SDCBP) (Figure 1b) (Kowal et al. 2016). Additionally, we confirmed expression of calnexin (CANX), a protein associated with the endoplasmic reticulum, in peripheral mononuclear cell (PBMC) lysates. The absence of CANX in EV samples confirms the purity of the EV preparation.

### 3.2 EV‐LYSG101 Loading

**FIGURE 2:**
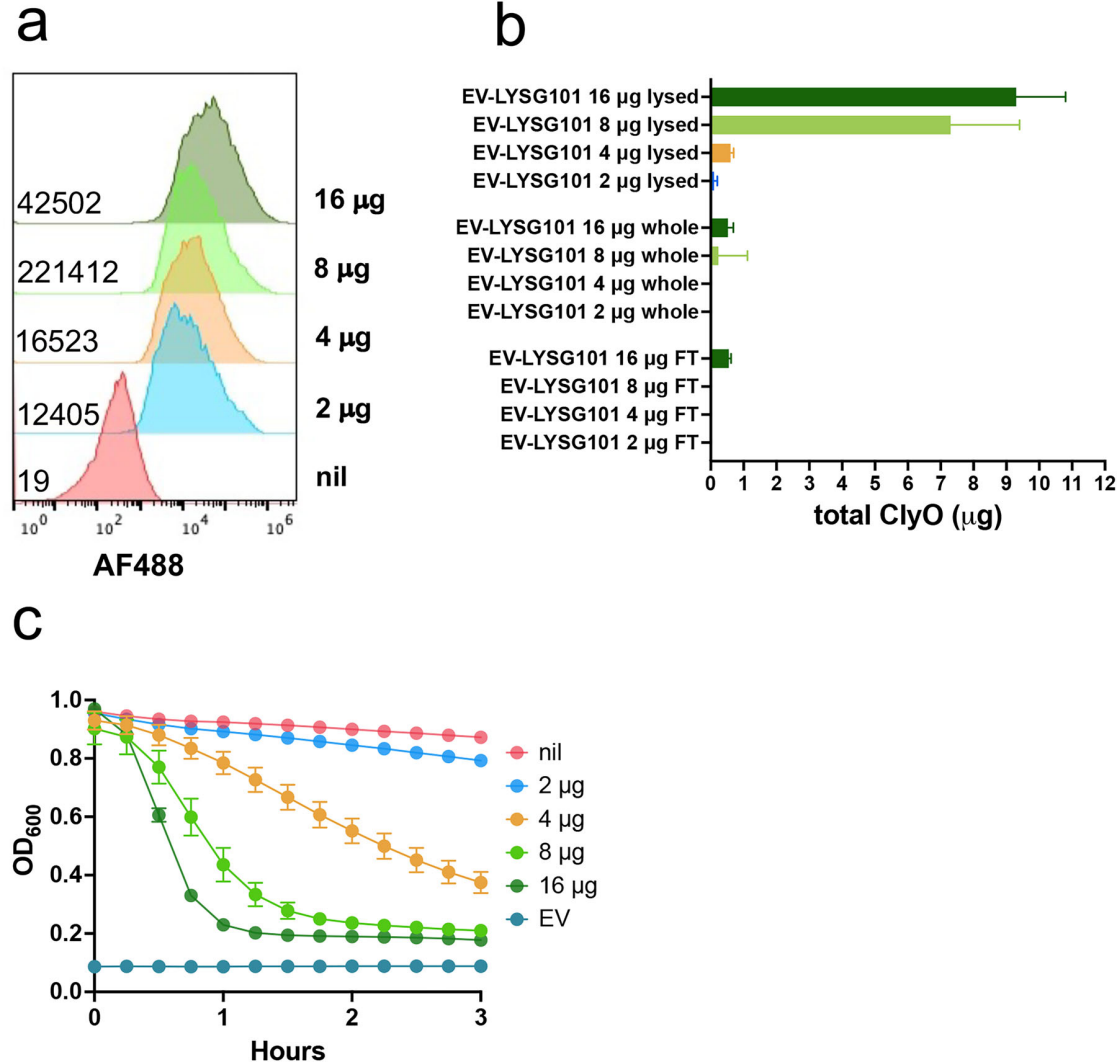
Verification of EV loading with LYSG101 and confirmation of EV‐LYSG101 lytic activity. (a) Flow cytometric analysis of EVs loaded with fluorophore‐labelled lysin. Increasing amounts of LYSG101‐AF488 were loaded into 1E9 serum‐derived EVs resulting in increasing AF488 signal (the geometric mean of the signal intensity is shown for each sample). (b) LYSG101 levels in 1E+9 EVs loaded with 2, 4, 8 and 16 µg LYSG101 measured by ELISA. Total LYSG101 inside of and on the surface of EVs (lysed), surface‐bound LYSG101 (whole) and LYSG101 present in flow‐through (FT) from the post‐loading wash. (c) Kinetic analysis of *S. aureus* lysis by EV‐LYSG101. EV‐LYSG101 samples from (b) were incubated with *S. aureus* over 3 h, measuring culture turbidity (OD_600_) every 15 min. Increasing concentrations of LYSG101 loaded into EVs results in effective bacterial lysis (decrease in OD_600_). Representative data from two independent determinations, each executed in triplicate. Error bars represent standard deviations.

EVs were loaded with four concentrations of LYSG101‐AF488 and counterstained with CellTrace Far Red to visualize EV‐associated lysin and not merely aggregates in solution (see gating scheme in Figure S1). EV‐LYSG101‐AF488 was analysed by flow cytometry, showing increased fluorescence concomitant with an increased amount of loaded protein. The geometric means of AF488 signal intensity were as follows; 16 µg = 42504, 8 µg = 22141, 4 µg = 16523, 2 µg = 12405 and nil = 19 fluorescence units (Figure 2a). In addition, the efficiency of loading (using unlabelled lysin) was confirmed by ELISA, showing that LYSG101 is mostly retained within the EVs (lysed fraction) and not on the exterior surface (whole sample), nor does it remain unincorporated (flow though—FT) (Figure 2b). The most efficient loading amount in 1E9 EVs is 8 µg, an amount at which 7 µg is found in EVs, with 0.2 µg located on the exterior surface, with no discernable lysin in the flow through (Figure 2b).

### 3.3 EV‐LYSG101 Lytic Activity

Having verified the presence of LYSG101 in EVs, we next tested whether the EV‐LYSG101 preparation has lytic activity against MSSA. For these experiments, 1E9 EVs were loaded with 2, 4, 8 and 16 µg LYSG101, washed and recounted before coculturing with MSSA (at a turbidity of OD_600_ = 1). Lysis was observed in a dose‐dependent manner, with the most efficient lytic capacity observed for EVs loaded with 16 µg of LYSG101, which resulted in 50% lysis in 30 min (Figure 2c). As all EV‐LYSG101 preparations are routinely recounted and sized we noted that there was no change in EV diameter with loading at any concentration. The average final concentration of EV‐LYSG101 particles per well was 3.1E7 ± 1E7, with a total of 1 µg encapsulated lysin.

A serial dilution series with free LYSG101 (from 100 to 1.5 ng/mL) illustrates the dynamic range of lysis over time, whereby a concentration of 100 ng/mL lyses 50% of approximately 5E8 organisms (OD_600_ = 0.5) in 30 min and 100% in 1.5 h (Figure S2).

### 3.4 Lysis Demonstrated by SEM

**FIGURE 3:**
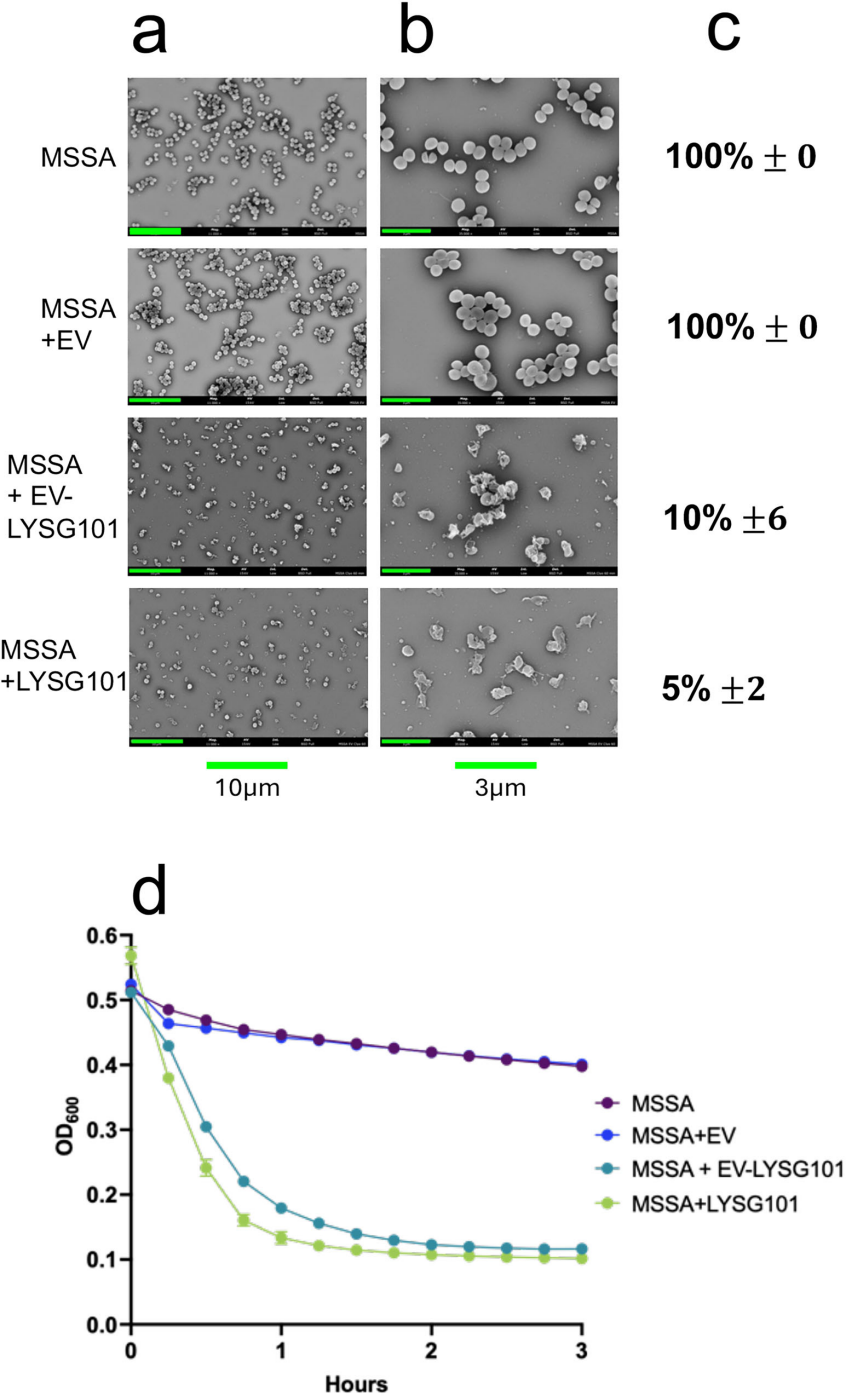
Scanning electron microscopic visualization of EV‐LYSG101 lytic activity. (a) MSSA alone, MSSA+EV particles (no lysin), MSSA+EV‐LYSG101, and MSSA+LYSG101 (no EVs) visualized at 10,000× magnification. (b) Samples visualized at 35,000× magnification. (c) Average percentage of intact whole cells (round shape) in five regions from the 10,000× images ± standard deviation. An average of 230 cells were examined per treatment. (d) Kinetic analysis of MSSA lysis by EV‐LYSG101 compared to 25 ng/mL free LYSG101 (i.e. not loaded into EVs). Representative data from three independent determinations, each executed in triplicate. Error bars represent standard deviations.

SEM was used to visualize the lytic effect of EV‐LYSG101 on MSSA. After 1‐h incubations, samples were imaged at 11,000× and 35,000× magnifications to assess changes in bacterial structural integrity. At each magnification, clusters of healthy coccal cells can be observed in both the untreated sample (MSSA alone) and in samples treated with EVs alone (i.e. no lysin); all (100%) of the cells appeared round/healthy (Figure 3a–c). Inversely, cocultures with EV‐LYSG101 and LYSG101 (25 ng/mL) alone (unencapsulated) yielded few structurally recognizable cells, 10%±6 and 5%±2 respectively (Figure 3a–c). The lytic effect is more clearly observed with the 35,000× magnification (Figure 3b), wherein bacteria appear ‘flattened’ and distorted in a non‐uniform manner, most likely due to the loss of cytoplasmic contents, as has been observed in other studies of lysin‐treated staphylococci (Vila‐Farres et al. 2023).

### 3.5 Variability of LYSG101 Loading Into EVs From Different Donor or Cellular Sources

**FIGURE 4:**
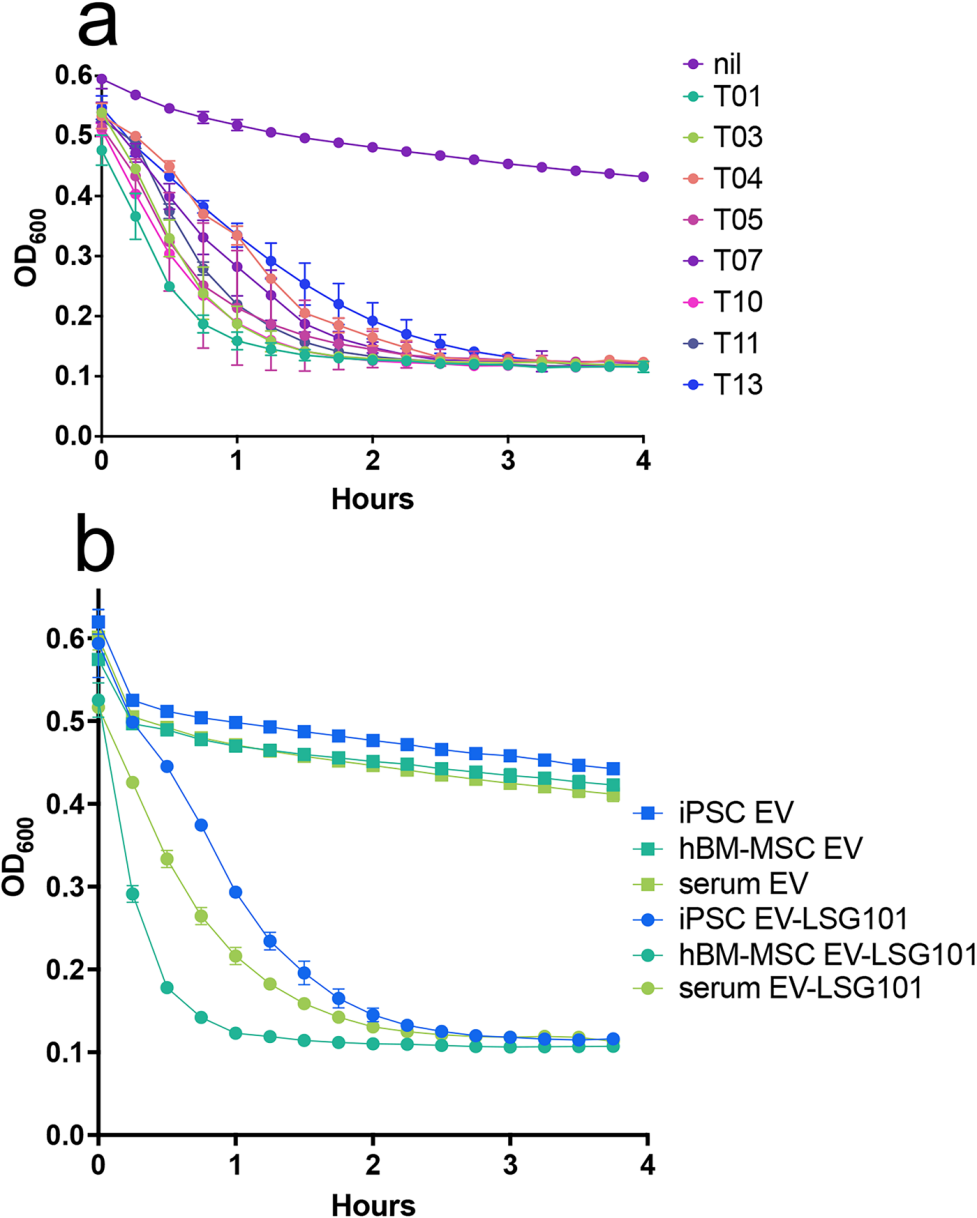
Lytic activity of LYSG101 loaded into EVs from different sources. (a) Kinetic analysis of MSSA lysis by LYSG101 loaded into EVs isolated from the serum of eight different donors (T01–T13). (b) Kinetic analysis of MSSA lysis by LYSG101 loaded into EVs purified from donor serum and obtained from commercial sources—induced pluripotent stem cell (iPSC) EVs and human bone marrow mesenchymal stem cell (hBM‐MSC) EVs. Representative data from two independent determinations, each executed in triplicate. Error bars represent standard deviations.

Human serum‐derived EVs (1E9) from eight different human donors were loaded with 8 µg LYSG101 and incubated with MSSA in a kinetic lysis assay. We observed variability in initial lysis efficiency, however, all preparations lysed MSSA by 3.5 h of incubation (Figure 4a). To further explore potential variability in the lytic activity of EV‐LYSG101 related to the source of EVs used, we compared serum‐derived EVs to those from induced pluripotent stem cells (iPSC, 65 nm diameter) and human bone marrow‐derived mesenchymal stem cells (hBM‐MSC, 149 nm diameter). We loaded 1E9 EVs (from each source) with 8 µg LYSG101 and examined relative lytic activities. Native EVs had no lytic activity, whereas the lytic efficiency of EV‐LYSG101 was highest for the hBM‐MSC sources, followed by the serum‐derived source and the iPSC derived source (Figure 4b). A correlation was observed between improved lytic activity and larger EV diameters, which were 149, 89 and 65 nm for the hBM‐MSC, serum donor and iPSC EVs, respectively.

### 3.6 EV‐LYSG101 Activity Across Staphylococcal Isolates

**FIGURE 5:**
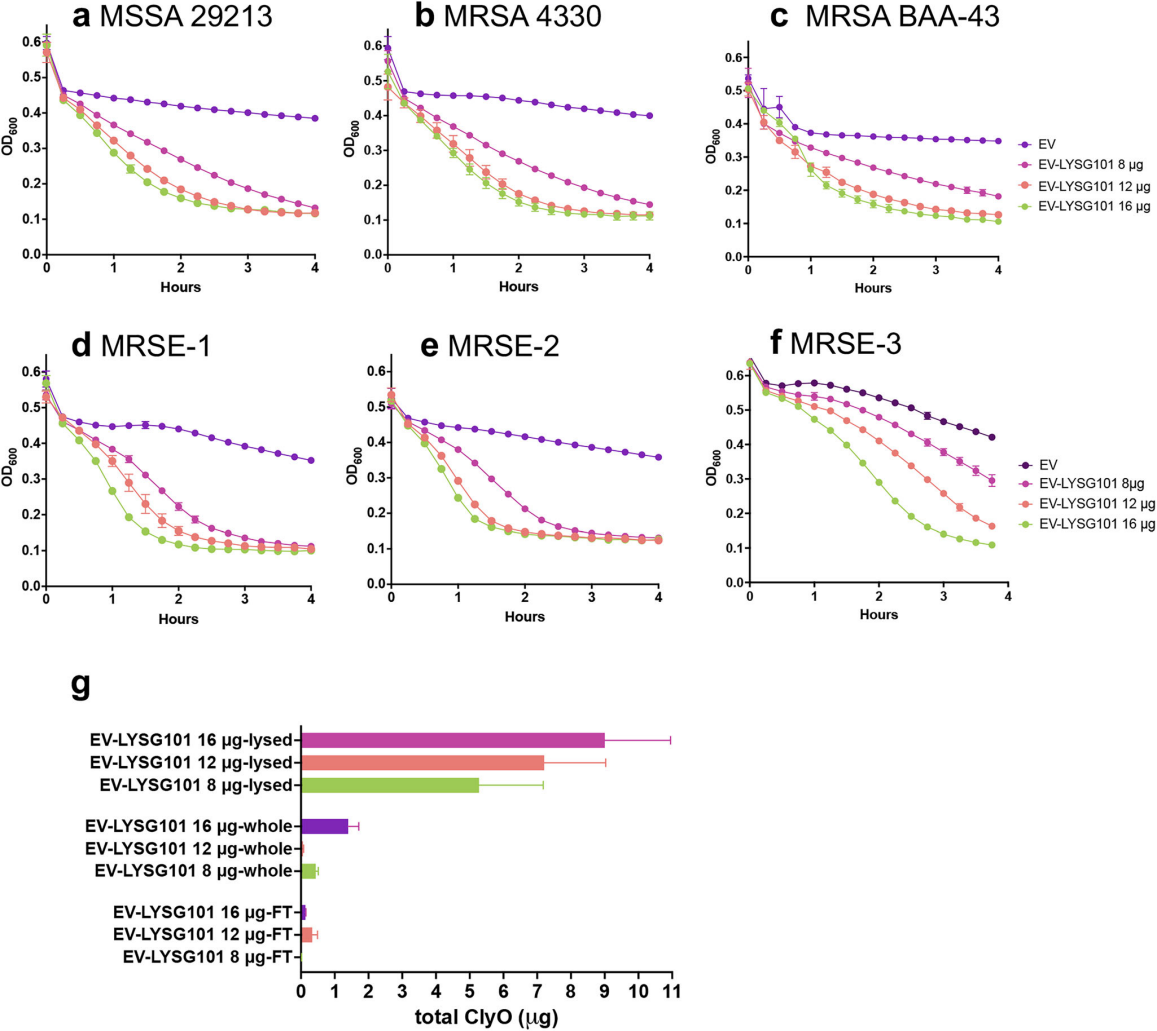
Kinetic analysis of Staphylococcal strains’ lysis by dose dependent EV‐LYSG101. (a–c) *S. aureus* strains. (d–f) *S. epidermidis* strains. (g) Evaluation of LYSG101 loading efficiency by ELISA. Total LYSG101 inside of and on the surface of EVs (lysed), surface‐bound LYSG101 (whole) and LYSG101 present in flow‐through (FT) from the post‐loading wash. Representative data from two independent determinations, each executed in triplicate. Error bars represent standard deviations.

Antibiotic resistance is a major obstacle in eradicating life‐threatening infections. Three strains of *S. aureus* (one MSSA and 2 MRSA strains, Figure 5a–c) and three strains of S. *epidermidis* (MRSE, Figure 5d–f) were tested for sensitivity to EV‐LYSG101. For these experiments, 1E10 EVs were loaded with three concentrations of lysin (8, 12 and 16 µg) and incubated with each bacterial isolate for 4 h. All three *S. aureus* isolates were completely lysed by 3 h, as were two of the three *S. epidermidis* isolates (MRSE‐1 and MRSE‐2, Figure 5d,e). While the MRSE‐3 isolate was less sensitive, complete lysis was achieved using EV‐LYSG101 (loaded with 16 µg) by 4 h (Figure 5f). EV‐LYSG101 was likewise active against *Staphylococcus lugdunensis* but demonstrated no activity against an *E. coli* control strain (Figure S2).

The concentration of LYSG101 in the EV samples was tested by ELISA prior to the lytic assay, showing that there is a limit to how much lysin can be packaged (Figure 5g). For example, when loading 1E10 EVs with 8 µg LYSG101, 5.2 µg remained internalized after the cleanup procedure, whereas loading with either 12 or 16 µg resulted in retention of 7.3 and 9 µg, respectively.

### 3.7 EV‐LYSG101 Disrupts MRSA Biofilm as Efficiently as Free Lysin

**FIGURE 6:**
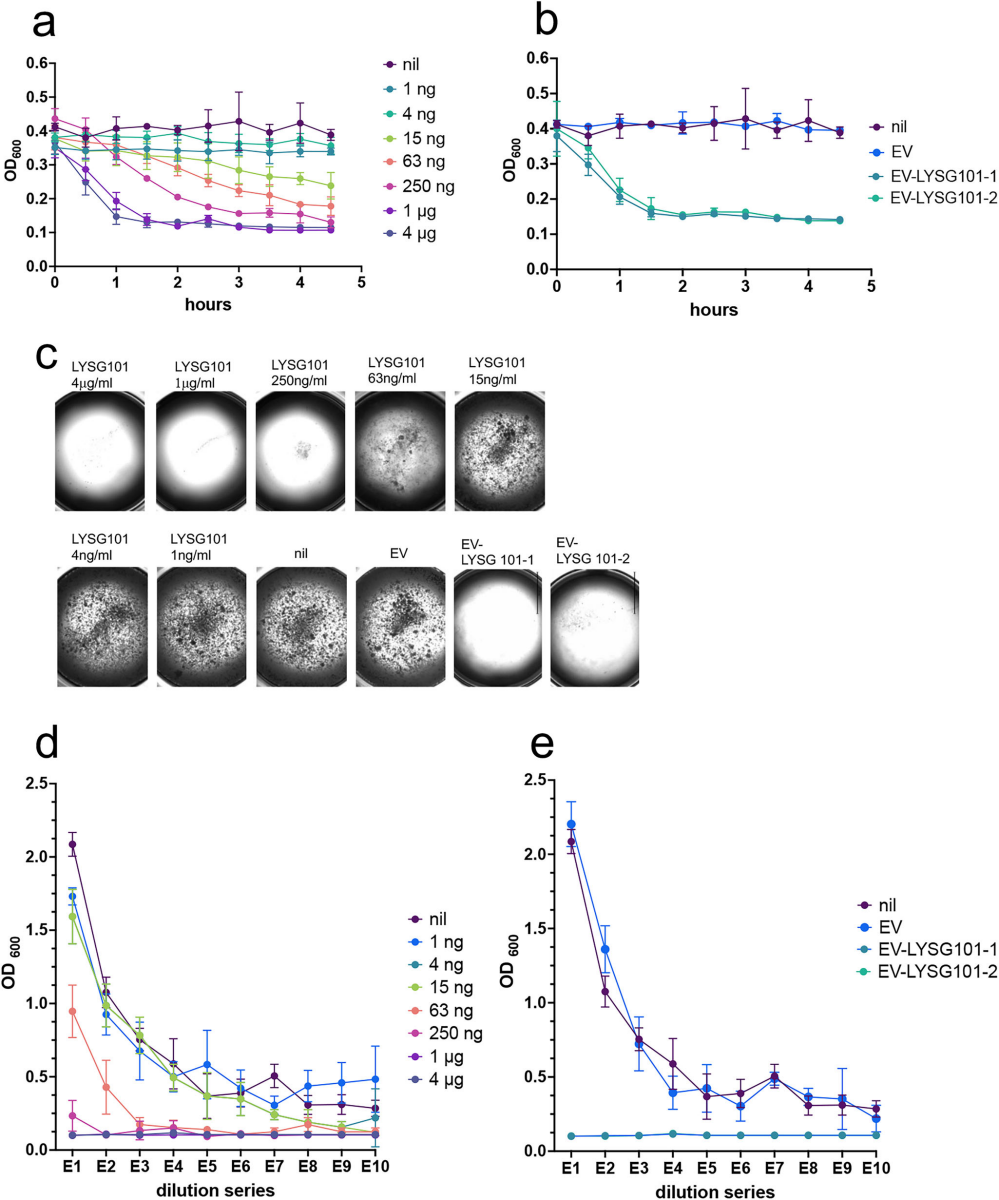
EV‐LYSG101 eradicates MRSA biofilm. (a) Kinetic analysis of MRSA BAA‐42 biofilm eradication with a serial dilution series of LYSG101. (b) Kinetic analysis of MRSA BAA‐42 biofilm eradication with two EV‐LYSG101 preparations. (c) Microscopic images (4×) of wells after the 4.5 h incubation with LYSG101 or EV‐LYSG101 from (a) and (b). (d) OD_600_ measurements of ten‐fold dilution series (E1‐E10) of residual bacterial cells in each well after LYSG101 treatment in (a). (e) OD_600_ measurements of ten‐fold dilution series of residual bacterial cells in each well after EV‐LYSG101 treatments in (b). Representative experiment of two independent determinations each executed in quadruplicate. Error bars represent standard deviations.

Staphylococcal biofilms are a primary driver of antibiotic failure in chronic infections (Tran et al. 2023). We therefore tested whether EV‐LYSG101 can eradicate biofilm bacteria. Biofilms formed by MRSA strain ATCC BAA‐42 were formed over 24 h and treated with a serial dilution range of LYSG101 (unencapsulated) and a fixed concentration of either of two EV‐LYSG101 preparations (1E9 EVs containing 230 ng LYSG101 per well). As above, OD_600_ was monitored over 4.5 h. Unencapsulated LYSG101 at the concentrations of 4 µg, 1 µg and 250 ng/mL completely eradicated the biofilm (Figure 6a). Both EV‐LYSG101 preparations eradicated biofilm in a manner equivalent to that observed using unencapsulated LYSG101 at 250 ng/mL (Figure 6b).

Visualization of the treatments immediately after the anti‐biofilm assay show no identifiable bacteria in wells containing either free LYSG101 (tested at 4 µg, 1 µg and 250 ng/mL) or the EV‐LYSG101 preparations (Figure 6c). Optical density measurements likewise confirmed the absence of bacteria from these treated samples (Figure 6d,e).

### 3.8 EV‐LYSG101 Stability

**FIGURE 7:**
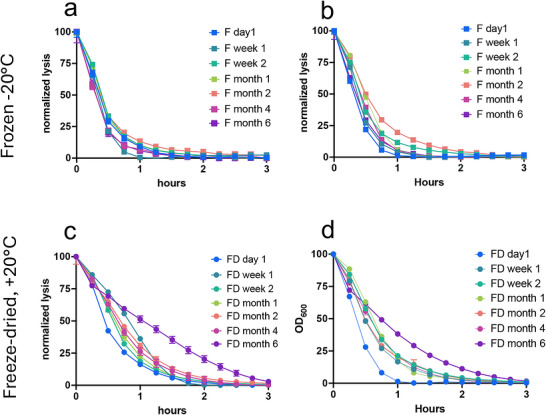
Stability assay comparing the effect of frozen versus freeze‐dried storage conditions on EV‐LYSG101 lytic function over time. (a) and (c) EV‐LYSG101‐1. (b) and (d) EV‐LYSG101‐2. (a) and (b) Frozen (F) EV‐LYSG101 stored at ‐20°C. (c) and (d) Freeze‐dried (FD) EV‐LYSG101 stored at room temperature 20°C (FD). OD reduction curves from seven different time points were normalized and shown on a single graph per sample and storage condition. EV‐LYSG101 concentration was 3E10/well. Shown are two independent determinations each done in triplicate. Error bars represent standard deviations.

As our goal is to ultimately manufacture shelf‐stable EV‐LYSG101, we initially examined stability of the compound, using the lytic assay format, for samples either stored frozen (F) at −20°C (Figure 7a) or stored at room temperature after freeze drying (FD) (Figure 7b). For this study, 6E+11 EVs were isolated from serum of two donors (donor 1 in Figure 7a,c, donor 2 in Figure 7b,d) and loaded with 96 µg LYSG101 (EV‐LYSG101‐1 and EV‐LYSG101‐2); half of each sample was then aliquoted and frozen (−20°C) (Figure 7a,b) and the other half was aliquoted and freeze‐dried followed by room temperature storage (+20°C) (Figure 7c,d). LYSG101 activity was then measured in the lytic assay format after the indicated incubation times. For the assays, final concentrations of 3E9 particles/well were used, containing 1.2 µg of LYSG101. While complete lysis was delayed by 30 min in the freeze‐dried samples, no significant differences in lytic activity were discernable between the frozen and freeze‐dried groups at 4 months of storage. At 6 months, a reduction in lytic efficiency in the freeze‐dried samples (maximal lysis was delayed by 1 h) was observed (Figure 7c,d).

## 4 Discussion

We are developing serum‐derived EVs as a vehicle to deliver the anti‐staphylococcal lysin LYSG101 as part of a broader strategy to improve stability and delivery of lysin therapeutics, with PJI representing one motivating application for this platform. Lysins represent a potent and new antimicrobial biotherapeutic class, differentiated from antibiotics with respect to structure and mechanism of action. The antibiofilm effect of lysins, in particular that of LYSG101 (Xiao et al. 2026), is significant considering the critical role of biofilm in chronic PJI and antibiotic failures.

Clinical translation of lysin technology has heretofore been hampered by challenges related, in part, to reduced stability and activity in physiologically relevant environments. For this reason, protein engineering strategies have been used to generate lysin derivatives that are stable and active in blood, synovial fluid, and respiratory fluid (Seijsing et al. 2018; Grishin et al. 2019, Abdelkader et al. 2022). The anti‐staphylococcal lysin LYSG101 is one such engineered lysin, optimized for human medicinal use. The choice of clinical indication and trial design is likewise critical for development, and for this reason, intra‐articular dosing in a multi‐day format in PJI is attractive, to avoid problems associated with the exebacase bacteremia trial, in which a single intravenously administered dose was used (Fowler et al. 2020, 2024). Intra‐articular injection offers the potential for higher local concentrations in a confined compartment (i.e. the knee or hip joint) to better leverage the anti‐biofilm effect in a manner already demonstrated in compassionate use for staphylococcal PJI (Ferry et al. 2021, 2022).

To further support clinical implementation of lysins we now describe initial studies to develop a delivery system for LYSG101 based on EVs—lipid nanovesicles derived from human tissues and used for their ability to be loaded with and to transport therapeutic molecules like proteins and nucleic acids as treatments in cancer, regenerative medicine and infectious disease (Kumar et al. 2024). Their potential as delivery vehicles lies in their low immunogenicity, ability to deliver drugs to target sites, stability, sustained release of loaded cargo, and the capacity for intracellular delivery. These features address many of the current limitations for lysins.

Initial in vitro proof‐of‐concept studies with EV‐LYSG101 are described in this report. EVs were isolated from multiple human donors, showing a consistent size distribution and expression of EV markers, such as CD9, CD63 and SDCBP. The exclusion of endoplasmic reticulum protein CANX, confirms that particle preparations were free of cellular debris or apoptotic bodies. The efficiency of protein loading into EVs was demonstrated using fluorescently‐tagged LYSG101 and measured by flow cytometry. Loading was independently confirmed by ELISA, with minimal detection of surface‐associated or unincorporated LYSG101. For EV preparations of 10E10 particles, a potential therapeutic dose for animal studies, we observed optimal loading at lysin concentrations of 16 ug/mL, with 9 µg of internalized protein (with 2%–3% of total protein on the surface); loading capacity was similar for EV preparations derived from a range of human serum donors. Not surprisingly, larger‐sized particles, such as hBM‐MSC EVs and donor serum‐derived EVs with diameters of 149 and 89 nm, respectively, packaged more LYSG101 than smaller iPSC EV with a diameter of 65 nm.

We furthermore demonstrated dose‐responsive killing of MSSA by EV‐LYSG101 in the lytic assay format. Here, 50% lysis of the target cell culture (5E8 CFU) was achieved within 30 min in the presence of 3.1E7 ± 1E7 particles (encapsulating an estimated total amount of 1 µg of lysin); equivalent kinetics were observed using 100 ng/mL concentration LYSG101 alone (i.e. ‘naked protein’). The killing effect of EV‐LYSG101 observed by SEM was identical to that using LYSG101, consistent with osmotic lysis and loss of cytoplasmic contents as has been observed for other lysins (Vila‐Farres et al. 2023). Lytic activity for EV‐LYSG101 was additionally observed against both MRSA and MRSE isolates in addition to *S. lugdunensis* (but not *E.coli*). Target specificity (for *Staphylococcus* species) and an absence of cross‐resistance with antibiotics (i.e. activities demonstrated against both methicillin‐sensitive and ‐resistant isolates) are critical features for lysins, demonstrated here and to be further examined in development. Overall, these results show that LYSG101 (a 28.6 kDa lysin protein) can be efficiently packaged into human serum‐derived EVs and retain its function.

Clinical translation will depend on the anti‐biofilm activity encoded by lysins and LYSG101. Staphylococcal biofilms are a significant factor in PJIs, typically forming on implant surfaces and resisting the activity of antibiotics and immune surveillance (Shoji and Chen 2020; Taha et al. 2018; Visperas et al. 2022). For this reason, it is of critical importance that we demonstrate an anti‐biofilm effect for EV‐LYSG101 against a prolific biofilm‐forming MRSA strain, previously used in the literature for other anti‐biofilm lysins (Schuch et al. 2017). The complete eradication of biofilm bacteria at high concentrations (OD = >1) over 4.5 h by 1E9 particles (encapsulating an estimated total amount of 230 ng of lysin) was equivalent to the effect using the 250 ng/mL concentration of LYSG101 alone in an anti‐biofilm dilution series. The OD reduction‐based method described here (coupled with visual demonstration of disrupted biofilm), is intended only as initial proof‐of‐concept for the capacity to address bacterial biofilms, as has been more thoroughly demonstrated for LYSG101 (Xiao et al. 2026) and for other lysins both in vitro (Schuch et al. 2017; Shahrour et al. 2025) and in vivo (Souche et al. 2022; Kebriaei et al. 2021; Shah et al. 2020; Alreja et al. 2024; Swift et al. 2021). The potent anti‐biofilm effect that will be further studied throughout development includes the context of animal PJI models.

Lastly, we show that EV‐LYSG101 retains its function when stored long‐term at ‐20°C (as a frozen liquid) and at room temperature (as a lyophilized powder for reconstitution). A shelf‐stable compound will be an important concern for future formulations in Chemistry, Manufacturing, and Control processes as we progress EV‐LYSG101 through the pre‐clinical pathway to develop this novel modality for the PJI indication in critically ill patients.

Overall, this study provides an initial in vitro proof‐of‐concept showing (1) the ability to encapsulate anti‐staphylococcal lysin in EVs in a stable form, and (2) the capacity of these EV‐lysins preparations to deliver potent antimicrobial effects against planktonic and biofilm bacteria. Further development is now warranted to assess the benefits and utility of encapsulation in overcoming many of the cited challenges facing lysins in clinical translation, with respect, particularly to immunogenicity and stability. Included in this work will be elucidation of the mechanism for antimicrobial action of EV‐lysins. It is currently envisioned that lysins are either slowly or rapidly released from EVs in the presence of bacteria in a process that could involve direct contact and subsequently high local lysin concentrations at the cell wall target; the rapid and potent antimicrobial effect observed here, taken with the otherwise stable nature of EV‐lysin particles suggests that a direct interaction does mediate release, however, this remains to be shown. Other future directions are described below.

### 4.1 Future Directions

#### 4.1.1 Optimize and Standardize Loading of LYSG101 Into Commercially Available MSC Derived EVs

To avoid donor‐dependent variability of the EV‐LYSG101 complex, we will standardize loading and function using hBM‐MSC. In addition, we will examine levels of endogenous proteases and include storage stability experiments. This complex will be used in a rat PJI mode, as outlined below.

#### 4.1.2 Measure EV‐LYSG101 Uptake by Bacteria

It is understood that bacteria can fuse with bacterial EVs (BEVs), produced by other bacteria and that this fusion can result in the delivery of proteins or DNA (Berleman and Auer 2013; Liu et al. 2018, 2019; Mobarak et al. 2024). BEVs can also be taken up by human cells through a number of endocytic pathways (Amabebe et al. 2024; Brakhage et al. 2021). Biological functions of EVs in host‐microbe interactions focuses in the sequelae of BEVs uptake by mammalian cells (reviewed by Fang et al. 2022), but little is known about the converse scenario: do bacteria take up human EVs? It is possible that EV‐LYSG101 fuses with bacterial cells to directly deliver lysin to surface peptidoglycans, resulting in cell wall destabilization and lysis. Conversely, LYSG101 attached to the EV surface membrane may initiate lysis followed by the release of the lysin cargo.

#### 4.1.3 Measure EV‐LYSG101 Activity Against Intracellular Staphylococci

Intracellular targeting of staphylococci is an important driver for using the EV delivery technology, compared to lysin alone. For this reason, the ability of EVs to deliver LYSG101 to efficiently kill *S. aureus* internalized within osteoblasts will be examined using a previously developed intracellular activity assay (Kolenda et al. 2020).

#### 4.1.4 Testing EV‐LYSG101 in the Rat PJI Model

The successful demonstration reported here of in vitro anti‐staphylococcal activity for EV‐LYSG101 is to be followed by a tolerability and efficacy study using a relevant rat model of *S. aureus* PJI similar to that previously described (Morris et al. 2019) to determine biodistribution, tolerability, and therapeutic efficacy in vivo. A dose‐response study is envisioned for EV‐LYSG101 tested both alone and in addition to a standard‐of‐care antibiotic. Comparator arms will include the testing of LYSG101 alone. Studies will be done to evaluate EV‐LYSG101 activity in physiologically relevant matrices such as human serum and synovial fluid to better model the inflammatory environment of PJI.

#### 4.1.5 Testing Immunogenicity of EV‐LYSG101

Because repeat intra‐articular dosing may be required in chronic implant‐associated infections, future studies will evaluate immunogenicity of EV‐LYSG101, including antibody responses to both EV components and lysin cargo following repeated exposure.

## Author Contributions

**Dominique B. Hoelzinger**: conceptualization, investigation, writing – original draft, writing – review and editing, formal analysis, project administration, methodology, data curation. **Ava M. Koehler de Celaya**: methodology, data curation, investigation. **Cheryl E. Myers**: methodology, investigation, writing – review and editing. **Natasha P. Dyal**: methodology, investigation. **Christopher F. Saling**: conceptualization, funding acquisition, writing – review and editing. **Raymond Schuch**: conceptualization, writing – review and editing. **Gina A. Suh**: conceptualization, funding acquisition, writing – review and editing.

## Conflicts of Interest

Raymond Schuch is an employee of Precisio Biotix Therapeutics, Inc. (Dover, DE).

## Supporting information




**Supplementary Figures**: jex270174‐sup‐0001‐Figures.docx

## Data Availability

The data that support the findings of this study are available from the corresponding author upon reasonable request.
