# Supplementary material for: EV‐LYSG101: Extracellular Vesicles Loaded With Lysin LYSG101 for Potent Anti‐Staphylococcal Activity
Source: J Extracell Biol. 2026 Aug 6;5(8):e70174. doi: 10.1002/jex2.70174 (PMC13448156; doi:10.1002/jex2.70174)
Supplement: Supplementary file 1 [file JEX2-5-e70174-s001.docx]

Supplemental figure legends for JEXB-2025-12-0139.R1

**Supplemental figure 1**. Gating scheme for EV flow cytometry. Unstained native EVs were used to set the gains and thresholds for detection of sub-micron particles (**a and b**). Single stains included CellTrace Far Red (CTFR) stained EVs to set parameters for the red channel (**c**) and EVs loaded with AF488 labeled LYSG101 to set parameters for the AF488 channel (**d**). EV-LYSG101-AF488 counterstained with CTFR were detected in (**e**) and the red oval depicts the gate used for the histograms shown in Figure 1.

**Supplemental figure 2**. Kinetic analysis of dose dependent lysis by free LYSG101 illustrates the dynamic range of lysis over time. The concentration of 100 ng/mL lyses 50% of approximately 5E8 organisms (OD_600_=0.5) in 30 minutes and 100% in 1.5 hours. LYSG101 was active against *S. aureus* both MSSA and MRSA , *S.epidermidis* ( one of three clinical isolates with identical results is shown) and *S. lugdunensis* but demonstrated no activity against an *E. coli* control strain.
